# Long-duration spaceflight adversely affects post-landing operator proficiency

**DOI:** 10.1038/s41598-019-39058-9

**Published:** 2019-02-25

**Authors:** Steven T. Moore, Valentina Dilda, Tiffany R. Morris, Don A. Yungher, Hamish G. MacDougall, Scott J. Wood

**Affiliations:** 1 School of Engineering and Technology, Central Queensland University, North Rockhampton, QLD Australia; 2 Human Aerospace Laboratory, Neurology Department, Icahn School of Medicine at Mount Sinai, New York City, USA; 3 School of Psychology, University of Sydney, Sydney, NSW Australia; 4 NASA Neuroscience Laboratory, Johnson Space Center, Houston, TX USA

## Abstract

Performance of astronaut pilots during space shuttle landing was degraded after a few weeks of microgravity exposure, and longer-term exposure has the potential to impact operator proficiency during critical landing and post-landing operations for exploration-class missions. Full-motion simulations of operationally-relevant tasks were utilized to assess the impact of long-duration spaceflight on operator proficiency in a group of 8 astronauts assigned to the International Space Station, as well as a battery of cognitive/sensorimotor tests to determine the underlying cause of any post-flight performance decrements. A ground control group (N = 12) and a sleep restriction cohort (N = 9) were also tested to control for non-spaceflight factors such as lack of practice between pre- and post-flight testing and fatigue. On the day of return after 6 months aboard the space station, astronauts exhibited significant deficits in manual dexterity, dual-tasking and motion perception, and a striking degradation in the ability to operate a vehicle. These deficits were not primarily due to fatigue; performance on the same tasks was unaffected after a 30-h period of sleep restriction. Astronauts experienced a general post-flight malaise in motor function and motion perception, and a lack of cognitive reserve apparent only when faced with dual tasks, which had recovered to baseline by four days after landing.

## Introduction

For future exploration-class missions to asteroids or other planetary bodies crewmembers will be required to perform operational tasks following extended periods of microgravity exposure. It is well documented that astronauts returning from long-duration missions to the International Space Station (ISS) exhibit adverse sensorimotor^1^, cardiovascular^2^ and neuromuscular^3^ effects upon return to Earth due to in-flight adaptation to microgravity. How these physiological changes affect post-landing operator proficiency is not well understood, but results from the shuttle era demonstrate that even short-duration missions adversely affect pilot performance. Our analysis of touchdown speeds for the first 100 missions^4^ demonstrated that 20% of orbiter landings were outside of acceptable limits, and the maximum speed of 217 kts (main gear tire limit) had been equaled or exceeded six times. The hardest touchdown on record (STS-90 at 224 kts) occurred following the commander’s momentary loss of orientation (‘tumbling the gyros’) after an active head movement just prior to touchdown^4^, and the second hardest touchdown (STS-3 at 220 kts) involved a pilot-induced oscillation after main-gear touchdown and prior to derotation (nose-gear touchdown)^4^.

Although no piloted landings have occurred following long-duration missions, the collision of the unmanned Progress 234 with the Mir space station in 1997 suggests that prolonged spaceflight can negatively impact operator performance. In this instance the commander, after 136 days on orbit, was tasked to remotely pilot the Progress from a distance of 6 km to dock with Mir. The collision was initially attributed to piloting errors in the form of ‘late realization that the closing rate was too high’ and ‘incorrect final avoidance maneuvering’^5^, although subsequent reviews determined that a variety of other factors contributed to the accident, such as fatigue (the commander’s request to withdraw from a sleep study due to chronic lack of sleep was refused by mission control prior to the collision)^6^, issues with the range radar (it was turned off for the final docking attempt at the behest of mission control), and inadequate planning and crew training^7^.There have been five significant teleoperation incidents aboard the ISS^8^, including a collision between the Space Station Remote Manipulator System (SSRMS, aka Canadarm2) and the shuttle payload door; and close calls between the SSRMS and an external UHF antennae, and between the SSRMS and the Shuttle Remote Manipulator System (SRMS, aka Canadarm), in which the two robotic arms crossed within 1.5 m of each other^8^.

What is the basis for post-mission decrements in operator proficiency? Most of the acute physiological effects associated with spaceflight occur at the gravitational transitions, from 1-g to 0-g at launch and 0-g back to 1-g during landing. For the first 24 to 72 hours in-flight and for several days post-flight crewmembers experience Space Adaptation Syndrome^9^, with motion sickness-like symptoms triggered by head movement. Two likely contributors are the shift of fluid to the upper body and head in microgravity and vestibular conflict. Astronauts returning from spaceflight are particularly prone to spatial disorientation; almost all shuttle crewmembers reported illusions of self- and/or surround-motion during active pitch and roll head movements during reentry and at wheels stop^10^. It is likely that post-flight spatial disorientation is due to in-flight deconditioning of otolith-mediated reflexes^11,12^, which on Earth maintain posture and gaze utilizing information from the otoliths regarding the relative orientation of the head to gravity. For example, pitch and roll head tilt on Earth generates both angular (semicircular canal) and linear (gravitational) acceleration (otolith) afferent signals from the vestibular endorgans that are integrated in the brainstem. In microgravity the same head motion produces only canal output, which conflicts with the expected response and proves provocative until a steady state is reached through adaptation to the microgravity environment, presumably by central reweighting of sensory input to reflect the predominance of angular information in the absence of gravity. During landing this process is reversed. Gravitational otolithic input is reintroduced, and during the period of readaption/reweighting to the gravitational field similar motion sickness symptoms occur as encountered in the first days of spaceflight.

In-flight studies have reported changes in fine motor control during manual tracking (joystick cursor control), indicative of an underestimation of the mass of the hand in microgravity^13^, decreased limb stiffness^14^, reduced peak velocity and acceleration in early stages of motion, maintenance of final accuracy by prolongation of the deceleration phase^14,15^, and a post-flight reduction in tracking gain^14^. Basic cognitive function has consistently proven to be unaffected by spaceflight in a number of studies^16^. Performance on simple and choice reaction time, Stroop interference, spatial processing^17^, memory search^18^ and mental rotation tasks^19^ was maintained in-flight and after landing. Results on dual tasking (performing manual tracking with a simultaneous distracting task) were less consistent. In-flight performance decrements in manual tracking with concurrent memory search were observed throughout a short-duration flight^20^ and for the first two weeks and for one week following a long-duration mission^21^. In contrast, tracking of targets with a parallel reaction time task were impaired on a shuttle mission in the same manner as on Earth^22^. Finally, in addition to microgravity, crewmembers are exposed to a variety of in-flight stressors that cumulatively could impact post-flight operator proficiency, such as altered light-dark cycle, sleep deprivation, elevated CO_2_ concentration, confinement, and high mental and physical workload.

Is operator proficiency even necessary? The space shuttle was capable of a fully automated landing (although never fully realized), and the Soviet shuttle (Buran) performed its only orbital flight and landing in 1988 without crew. The next generation NASA Earth-return spacecraft (Orion) plans to implement an Apollo-like water recovery of the crew module, although lunar and Mars landing craft may require some form of operator control or supervision. There is a compelling argument for maintenance of operator proficiency during and after spaceflight, even during automated tasks. The margin for error in the harsh environment of space is small, and in-flight failures of automatic control requiring corrective action from the crew have occurred in both the US and Russian space programs. Automated guidance of Voskhod 2 (1965) failed prior to reentry and the crew took control and manually positioned the spacecraft for reentry, selected the landing point and determined the correct timing and duration of the deorbit burn^23,24^. A manual retro-fire was also carried out on Soyuz 1 (1967) after on-orbit failure of the attitude control system led to an emergency return (ultimately unsuccessful due to failure of the parachute deployment pressure sensor)^23^. In 1966, a ‘stuck’ thruster in the attitude maneuvering system of Gemini VIII resulted in a roll rate approaching 60 rpm^25^. Although severely disoriented and with vision impaired^26^, the crew regained control of the spacecraft by disabling the attitude control system and engaging the reentry control system. An oxygen tank explosion in the service module during the 1970 Apollo 13 mission forced the crew to utilize the lunar module (LM) to maintain life support while shutting down the command module to preserve power for reentry^27^. The commander regained control of the combined service/command/lunar module stack using the LM thrusters to reestablish a slow roll to maintain thermal distribution. The crew, with support from mission control, performed a number of manually controlled burns with the LM descent engine (a contingency they had not trained for) to position the spacecraft for a successful return to Earth. More recently, Soyuz crew travelling to the ISS have twice disabled the automated docking system due to technical issues and performed manual docking with the ISS complex^28,29^.

This study was selected by NASA in 2009 to determine the impact of long-duration spaceflight on post-landing operator proficiency during seated tasks. Within 24-h of landing after an average of 171 days aboard the ISS, eight crewmembers were assessed with a cognitive/sensorimotor test battery and three full-motion simulations– driving a car, piloting a T38 jet, and navigation and docking of a Mars rover. In this report we present results from the test battery and car driving simulations.

## Methods

The experiments were approved by the Program for the Protection of Human Subjects at Icahn School of Medicine at Mount Sinai (study 08–1009) and the Institutional Review Board at NASA Johnson Space Center (protocol CR00000550), and all testing was performed in accordance with the relevant guidelines and regulations pertaining to the protection of human subjects as described in the Declaration of Helsinki and Health and Human Services 45 CFR 46. Subjects gave their written informed consent and were free to withdraw at any time. All test sessions were conducted in Building 266 at NASA Johnson Space Center (JSC), Houston, Texas.

Eight astronauts (all male), assigned to missions aboard the ISS from October 2012 until June 2015, participated in the flight study. Mean age was 47.5 years (SD 6.7; range 36–53), and time aboard the ISS ranged from 142 to 200 days (mean 170.8SD 20.4). In addition to the flight study, two groups of healthy subjects participated as ground-based controls; the ‘shadow’ group of twelve male subjects (mean age 39.0 years SD 9.7; range 25–58), and nine subjects (5 males; 4 females) in a sleep restriction group (mean age 40.0 years SD 10.6; range 26–57). All subjects held a valid driving license.

The astronaut subjects were tested four times pre-flight and three times post-flight. The first 90-minute session, scheduled on average 167.5 days (SD 62.6) prior to launch, was used to familiarize crewmembers with the cognitive/sensorimotor test battery and the driving simulations (data from these sessions were not analyzed). Baseline data were obtained from the subsequent three 60-min pre-flight sessions, which occurred 129.8 (SD 15.2), 82.1 (SD 10.5) and 73.8 (SD 10.0) days before launch. Crewmembers were tested at JSC on the day of return from the ISS (R + 0) approximately 20–22 h after touchdown in Kazakhstan, corresponding to late evening (10:00 pm – midnight) Houston time, following a ‘direct return’ from Karaganda aboard a NASA Gulfstream III aircraft. Due to mission constraints one subject was not available for testing until 7:00am Houston time the day after landing (approximately 30 h after touchdown). The mean gap between the final pre-flight test and the first post-flight session was 244.6 days (SD 14.5; range 217–267). The second and third post-flight sessions were conducted 4.1 days (SD 0.8; range 3–5) and 8.1 days (SD 1.2; range 6–10) after return (labelled R + 4 and R + 8, respectively).

Shadow testing was scheduled to closely mirror that of the astronaut subjects. Four baseline sessions (a 90-min familiarization followed by three 60-min baseline data collection sessions, analogous to the astronaut pre-flight schedule) were conducted with an average interval between sessions of 8.8 days (SD 1.1; range 7–12). The mean gap between the final baseline and first ‘post-gap’ session (G + 0, analogous to R + 0) was 244.8 days (SD 7.6; range 236–257), which was almost identical to the mean interval between the final pre-flight and first post-flight session for the astronauts (244.6 days). The second (G + 4) and third (G + 8) post-gap sessions were held 3.5 (SD 0.8; range 3–5) and 7.3 (SD 0.8; range 6–8) days after the G + 0 session.

Subjects participating in the sleep restriction group performed three 60-min baseline sessions (analogous to astronaut pre-flight testing) an average of 6.1 days (SD 2.4; range 4–10) apart; the first session was a combination familiarization/data collection session, the final two were data collection only. A week after the third baseline session (7.1 days SD 0.3; range 7–8) subjects participated in a ‘post-sleep deprivation’ session (S + 0, analogous to R + 0), following a 30-h sleep restriction protocol (see below).

### Cognitive/sensorimotor test battery

The test battery (Table 1) was initiated after the subject entered the test room and was seated at a desk. Computer-based tests were implemented in the LabVIEW G programming language (National Instruments Austin TX) running on a Sony Vaio PC laptop (reaction time, perspective taking, match to sample, manual tracking and dual tasking - the order of these tasks were randomized), or on the simulator control computer (motion perception).

**Table 1:** The cognitive/sensorimotor test battery.

Test	Methodology	Measures
Sleepiness Scale	Likert Scale	Subjective sleepiness (1–7)
Static Visual Acuity	Landolt ‘C’ Eye Chart at 10 ft	Acuity (logMAR)
Manual Dexterity	Purdue Pegboard Test	Number of pins placed in 30 s
Simple Reaction Time	Press left mouse button in response to screen icon	Reaction time (ms)
Perspective Taking	Indicate compass direction with respect to cockpit view	Time to complete, percent correct
Match to Sample	Indicate matching pattern from two similar possibilities	Time to complete, percent correct
Manual Tracking	Track onscreen target with mouse	Tracking error (pixels)
Dual Tasking	Respond to prompts to enter 4-digit codes while tracking	Tracking error (pixels); time to respond to dual task, percent correct response
Motion Perception	Indicate perceived gravitational vertical during random cabin tilt	Pitch and roll frequency response

The Stanford *Sleepiness scale* was used to quantify subjective changes in sleepiness^30^. Subjects were asked to choose an ordinal value from a list of statements that best described their state of sleepiness:

1. *Feeling active and vital*, *alert; wide awake*
2. *Functioning at a high level*, *but not at peak; able to concentrate*
3. *Relaxed; awake; not at full alertness; responsive*
4. *A little foggy; not at peak; let down*
5. *Fogginess; beginning to lose interest in remaining awake; slowed down*
6. *Sleepiness; prefer to be lying down; fighting sleep; woozy*
7. *Almost in reverie; sleep onset soon; lost struggle to remain awake*

*Static Visual Acuity* was assessed using a Landolt ‘C’ eye chart positioned 3.05 m (10 feet) away at eye level. Subjects indicated the orientation of the opening of the ‘C’ (left, right, up or down) along a line of 5 characters, typically starting at 10/10 (logMAR = 0). Visual acuity was determined as the smallest line on which the subject could correctly identify the orientation of at least 3 ‘C’ optotypes.

*Manual dexterity* was assessed with the Purdue Pegboard^31^ test (Lafayette Instrument, Lafayette IN). Subjects were tasked to place as many pins in a vertical row of slots (one at a time) within 30 s, first with the right hand, then with the left. The pins were then removed and subjects asked to place pairs of pins (with both hands simultaneously) in two vertical rows of slots within a 30 s period.

*Simple reaction time* was assessed by having the subject press the left mouse button as soon as possible after a circular icon appeared on a black screen^32^, based on the Simple Reaction Time task in the Automated Neuropsychological Assessment Metrics (ANAM –Vista Life Sciences, Washington DC). Preemptive presses were counted but ignored in the reaction time calculation. Total test time was 60 s.

A computerized *perspective taking* task^32^ was developed based on the Directional Orientation Test from the Test of Basic Aviation Skills, used by the US Air Force to assess potential pilot recruits^33^. In a previous study we found a significant adverse effect on perspective taking during Galvanic vestibular stimulation^32^, an analog of post-flight sensorimotor dysfunction in astronauts^34–36^. A topographical map was shown on the left of the screen with an aircraft icon at the center, heading in one of the four cardinal directions (north, south, east, or west). Subjects were instructed to imagine they were piloting this aircraft, and were asked to indicate a cardinal direction (e.g. ‘Which direction is East?’) relative to a larger aircraft image on the right-hand side of the screen using one of the four arrow keys. Participants performed 32 trials. Time to complete each task and number of correct responses were recorded.

Short-term memory for learned associations was assessed with the *match to sample* task^32^, based on the ANAM module. Subjects were instructed to memorize a single 4 × 4 array of blue and red squares presented for 2 seconds. After a 2-second delay two 4 × 4 patterns appeared on the screen, one of which matched the previously viewed array. Subjects were instructed to identify the matching pattern by pressing the corresponding right or left arrow key on the computer keyboard. The task consisted of 20 trials.

*Manual tracking* -  subjects were required to use the computer mouse with their dominant hand to maintain a cross-hair target inside a 15mm-diameter circle moving at 20 mm/s on the computer screen and randomly changing direction over a 60 s epoch^32^.

*Dual tasking* - subjects performed the tracking task above whilst responding to prompts from a second computer monitor for a 4-digit numerical code to be entered on a keypad with the non-dominant hand. The distracting task was performed continuously, and the time to respond and the number of correct responses were recorded, in addition to tracking performance.

**Figure 1:**
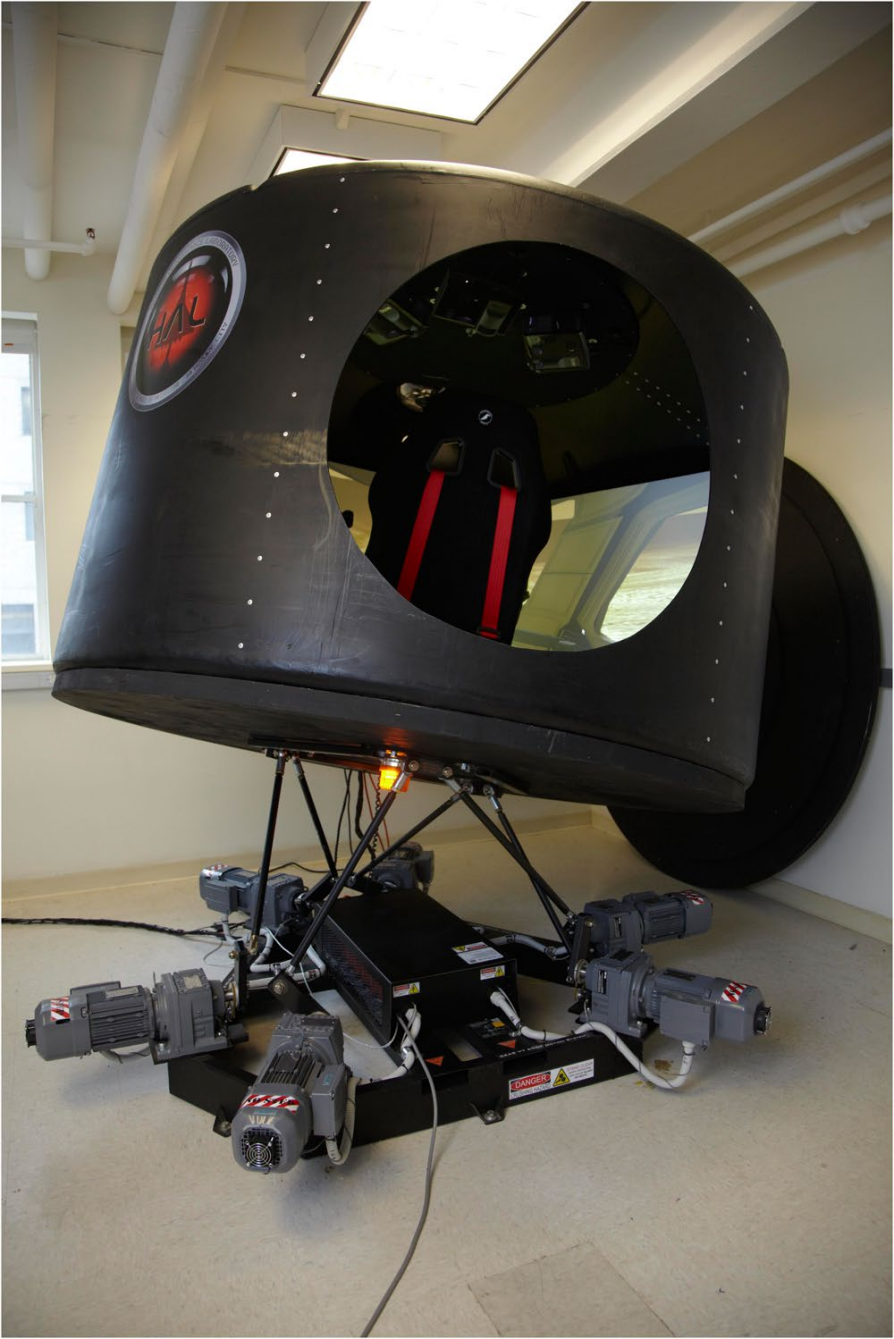
The 6 degree-of-freedom motion simulator.

The *motion perception* task was performed with the subject in the motion simulator (Fig. 1; described in the following section), seated and restrained with a harness and foam head-holder. Subjects were asked to close their eyes (verified verbally by the operator) and indicate gravitational vertical with the control stick while the cabin moved in a pseudorandom manner driven by a sum of seven sines with frequencies at 0.12, 0.25, 0.32, 0.43, 0.62, 0.80 and 0.98 Hz, first in roll for 60 s, then, after a short break, in pitch. Control stick orientation was acquired at 50 Hz throughout the period of motion.

### Motion simulator

The motion simulator (Fig. 1) was based on a 6 degree-of-freedom Stewart platform (V7, CKAS, Melbourne, Australia). A cylindrical polypropylene water tank (2.2 m diameter; 1.7 m height) formed the cabin, and was attached to a 50 mm thick plywood base bolted to the motion platform. Three ceiling-mounted short-throw digital projectors (BENQ 515ST) provided a 180° field-of-view display. Subjects were placed in a racing seat and restrained by a 4-point harness (Corbeau A4, USA). The control pod included a steering wheel (Trackstar 6000, ECCI, Mineapolis, MN) control stick (CH Products, Vista, CA), and pedals (Trackstar 6000, ECCI, Mineapolis, MN).

### Choice of simulation tasks

The major methodological constraints for this study were the time available for training the crew on the test battery and simulator tasks (90 minutes maximum per subject) and the duration of the critical R + 0 testing session (45 minutes total including the test battery and full-motion simulations). This restricted the number, duration and complexity of simulator tasks and necessitated an approach focusing on tasks on which the crew were already experienced (i.e., minimal training required). The investigators opted for a driving task (post-landing recovery of driving ability was of considerable interest to NASA’s medical branch), a T38 Talon landing simulation (the majority of astronaut subjects were veteran military test pilots with T38 experience), and, at NASA’s request, a Mars Rover simulation based on the prototype Space Exploration Vehicle^37^. In this report we describe the results from the driving simulations.

### Driving simulations

**Figure 2:**
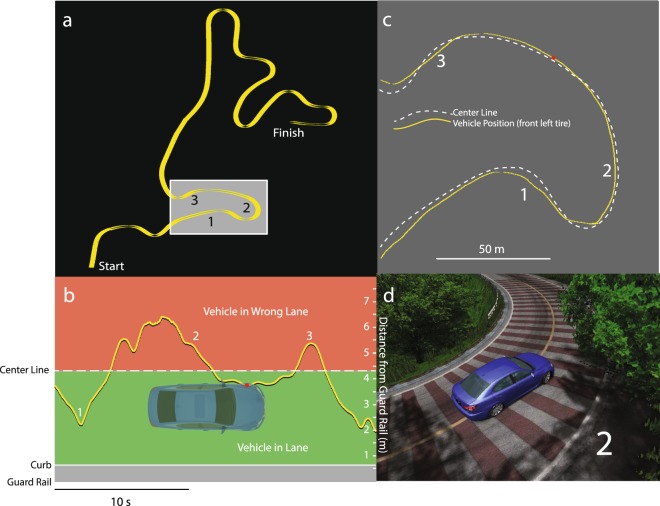
(**a**) The 3 km mountain course. (**b**–**d**) The position of the vehicle’s front left tire relative to the center line was acquired to determine deviations into the wrong lane.

All subjects performed a mountain road and cone course driving simulation, implemented using commercial software (rFactor, Image Space, Ann Arbor, MI). For the mountain course subjects were required to drive a car (Lexus ISF) along 3 km of twisting mountain road (Fig. 2a - based on an open source circuit ‘Harugahara’^38^) as quickly as possible while maintaining their position within the right lane. Data files were processed in LabVIEW to provide the position of the vehicle (left front tire) relative to the center line (Fig. 2b–d). The number of lane crossings, the time to correct (return to the right lane after each crossing) and the percentage of time spent in the wrong lane, were calculated, as well as mean and peak speed for each trial.

**Figure 3:**
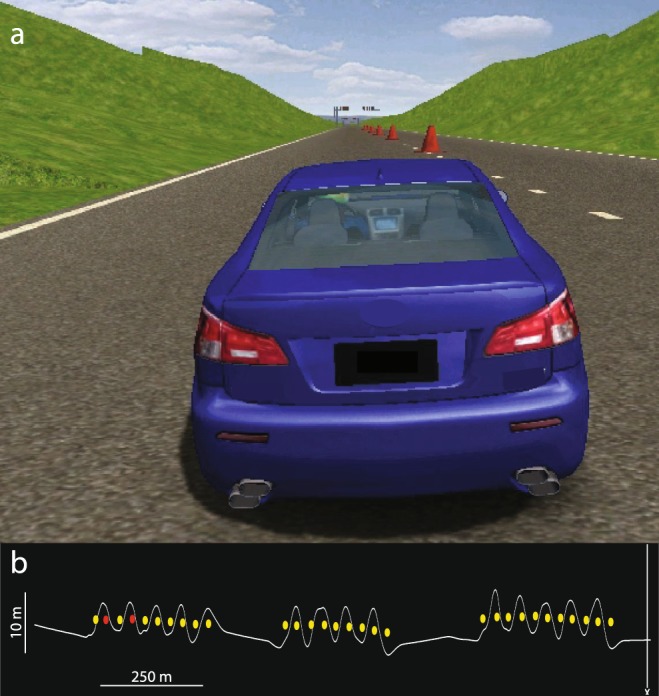
(**a**) Screenshot from the cone driving simulation. (**b**) Vehicle position data (white trace) and hit cones (red) from a trial.

The cone course was implemented using a commercially available track development tool (Bob’s Track Builder, bobstrackbuilder.net). Three sets of cones (numbering 10, 9, and 11 cones) were situated on a straight 1500 m section of road (Fig. 3). The space between cones averaged 30 m, and the distance between sets of cones was approximately 220 m. Subjects were instructed to drive as quickly as possible whilst slaloming around the cones without hitting them. The time to complete the course and the number of cones hit were calculated.

### Sleep restriction protocol

Sleep-restricted subjects performed a scaled-down protocol consisting of the sleepiness scale, manual tracking and dual tasking, and the driving simulations. Prior to the final ‘S + 0’ test session subjects in the sleep portion of the study underwent a 30-h period of sleep deprivation. At selected time points throughout the sleep restriction period, subjects participated in a short psychomotor vigilance test^39^ and recorded their sleepiness rating and activities in a worksheet on a tablet computer, the results of which were uploaded to a secure website to monitor compliance with the sleep restriction protocol. These tests were conducted at baseline when the subject awoke on the day before testing, at 5-h intervals for the first 10-h block, at 2-h intervals during the second 10-h block, and 1-h intervals during the final 10-h block, for a total of 18 tests. During the last 10 hours of sleep restriction subjects were asked to limit caffeine consumption, refrain from operating a vehicle or machinery, or participating in strenuous activities. The final vigilance test and worksheet at hour 30 were completed in the laboratory just prior to conducting the final ‘S + 0’ test session.

### Statistics

Due to the small data sets, a non-parametric single-tailed Mann-Whitney U test was implemented in SPSS (IBM Corporation, Armonk, NY) to determine if day of landing (R + 0) data changed significantly (p < 0.05) compared to preflight data; single-tailed as based on post-flight changes reported in the literature and our own previous experience, we hypothesized that long duration spaceflight would only impair astronaut function post-flight (i.e., we were not expecting improved performance on landing day compared to pre-flight).

## Results

**Figure 4:**
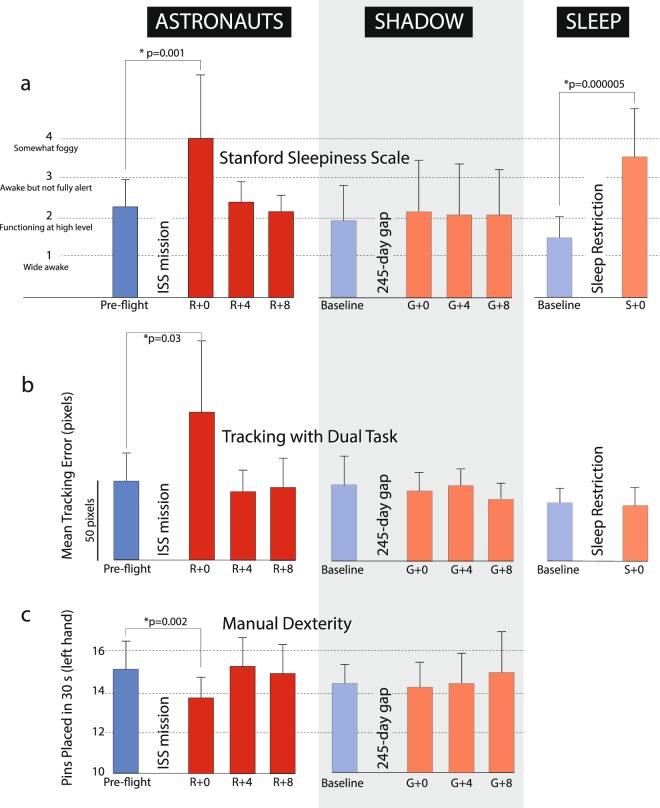
Results from the cognitive/sensorimotor test battery for the astronaut, shadow and sleep groups. (**a**) Subjective sleepiness rating. (**b**) Mean tracking error during dual tasking. (**c**) Manual dexterity (Purdue Pegboard test).

**Figure 5:**
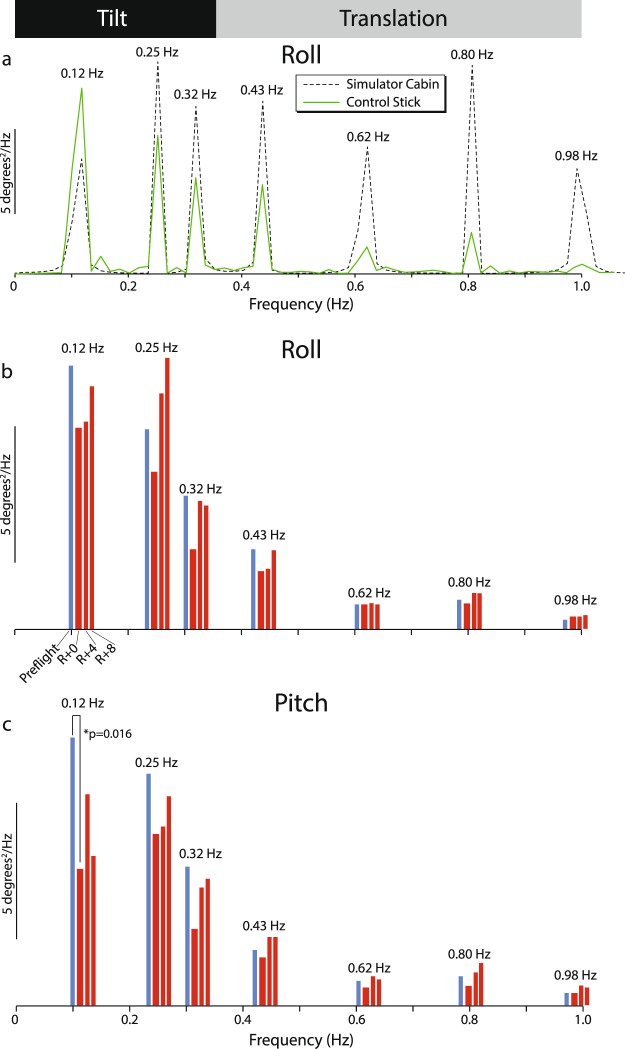
The motion perception task. (**a**) Power spectrum of roll cabin motion (dashed line – a sum of seven sinusoids) and subject response using the control stick to indicate gravitational vertical (green trace). The peak input response in roll (**b**) and pitch (**c**) at each of the seven frequencies.

No significant post-flight changes were observed in reaction time, perspective taking, match to sample, manual tracking or static visual acuity (Table 2). Crewmembers’ self-reported sleepiness was significantly higher (U = 30; p = 0.001) on landing day (R + 0) relative to the pre-flight baseline, and was comparable to the significant increase in sleepiness (U = 12; p = 0.000005) observed in the sleep group after the 30-h sleep restriction protocol (Fig. 4a). Although there was no increase in error on R + 0 during the manual tracking task alone, when a distracting (dual) task was added there was a significant increase (U = 43; p = 0.03) in mean tracking error in the astronaut group, which was not observed in the shadow or sleep groups (Fig. 4b). The astronauts alone also exhibited a small but significant decrease in manual dexterity on R + 0 for the left (non-dominant) hand (U = 33; p = 0.002; Fig. 4c), and the reduction in inserted pins was just above the threshold of significance for the right hand (U = 63.5; p = 0.08) and for both hands simultaneously (U = 60; p = 0.06). These post-flight changes in the astronaut group had returned to pre-flight levels by four days after landing (R + 4; Fig. 4). The astronauts exhibited a significantly reduced response (U = 47; p = 0.016) to pitch cabin motion at the lowest frequency (0.12 Hz) on landing day during the motion perception task (Table 3 and Fig. 5), and there was a tendency towards a blunted response on R + 0 in both pitch and roll for frequencies of 0.43 Hz and below. There was no consistent changes in roll or pitch perception in the shadow group (Table 3).

**Table 2:** Results from the cognitive/sensorimotor test battery. Significant changes shown in boldface.

ASTRONAUTS	PRE-FLIGHT	R + 0	R + 4	R + 8
Sleepiness Scale	2.13 (0.61)	**4.0 (1.6)**	2.38 (0.51)	2.14 (0.38)
Reaction Time (ms)	291.6 (14.2)	287.8 (19.6)	286.1 (15.5)	298.3 (36.8)
Perspective Taking (accuracy, %)	97.0 (1.7)	95.8 (4.6)	95.8 (7.6)	97.4 (2.4)
Perspective Taking (response time, s)	1.52 (0.24)	1.53 (0.31)	1.45 (0.30)	1.35 (0.27)
Match to Sample (accuracy, %)	95.3 (4.1)	93.2 (6.9)	96.0 (5.1)	98.9 (2.1)
Match to Sample (response time, s)	1.47 (0.27)	1.38 (0.23)	1.41 (0.33)	1.34 (0.25)
Tracking (mean error, pixels)	40.6 (26.3)	36.9 (11.6)	36.7 (19.7)	29.4 (7.1)
Dual Task Tracking (mean error, pixels)	48.4 (16.1)	**92.5 (49.2)**	43.0 (13.5)	45.7 (18.2)
Dual Task Input (response time, s)	5.38 (1.65)	5.83 (2.32)	6.07 (2.40)	5.20 (1.89)
Dual Task Input (accuracy, %)	94.1 (8.5)	94.5 (9.0)	93.8 (12.4)	95.0 (11.7)
Pegboard (Right Hand, # pins)	15.46 (2.48)	14.25 (1.49)	15.38 (1.41)	15.00 (2.31)
Pegboard (Left Hand, # pins)	15.13 (1.42)	**13.38 (1.06)**	14.75 (1.39)	14.57 (0.98)
Pegboard (Both Hands, # pins)	12.58 (1.84)	11.38 (1.41)	12.25 (1.39)	11.57 (0.53)
Static Visual Acuity (logMAR)	−0.17 (0.12)	−0.11 (0.13)	−0.16 (0.13)	−0.13 (0.13)
**SHADOW**	**BASELINE**	**G + 0**	**G + 4**	**G + 8**
Sleepiness Scale	1.90 (0.97)	2.17 (1.34)	2.08 (1.33)	2.08 (1.16)
Reaction Time	313.2 (41.8)	322.3 (33.3)	310.9 (12.9)	306.1 (13.3)
Perspective Taking (accuracy)	97.1 (5.4)	96.6 (4.4)	99.1 (1.0)	99.1 (1.4)
Perspective Taking (time to respond)	1.64 (0.38)	1.65 (0.31)	1.53 (0.29)	1.44 (0.27)
Match to Sample (accuracy)	92.8 (6.4)	93.2 (7.6)	96.8 (4.7)	96.6 (3.9)
Match to Sample (time to respond)	1.56 (0.44)	1.62 (0.43)	1.47 (0.30)	1.42 (0.31)
Tracking (mean error)	32.8 (30.0)	29.2 (9.1)	29.9 (8.8)	27.7 (9.8)
Dual Task Tracking (mean error)	47.6 (17.9)	43.2 (11.6)	45.5 (10.2)	38.0 (9.3)
Pegboard (Right Hand)	14.92 (2.17)	14.75 (2.26)	14.83 (2.76)	15.00 (2.41)
Pegboard (Left Hand)	14.33 (2.17)	14.08 (1.83)	14.25 (2.06)	15.00 (1.21)
Pegboard (Both Hands)	11.61 (1.13)	11.67 (1.37)	12.00 (1.83)	11.83 (1.53)
Static Visual Acuity	−0.23 (0.06)	−0.19 (0.10)	−0.18 (0.10)	−0.20 (0.11)
**SLEEP**	**BASELINE**	**S + 0**		
Sleepiness Scale	1.5 (0.6)	**3.6 (1.2)**		
Tracking (mean error)	25.9 (8.0)	23.4 (3.1)		
Dual Task Tracking (mean error)	35.9 (9.0)	33.9 (11.5)

**Table 3:** Results from the motion perception task (peak power). Significant changes shown in boldface.

Frequency (Hz)		0.12	0.25	0.32	0.43	0.62	0.80	0.98
**ASTRONAUTS**								
Roll	Pre-flight	9.4 (4.4)	7.1 (3.0)	4.7 (2.0)	2.0 (1.2)	0.81 (0.62)	1.1 (1.0)	0.33 (0.38)
	R + 0	4.2 (1.9)	3.2 (1.2)	1.9 (1.3)	1.6 (1.3)	0.41 (0.40)	0.53 (0.66	0.33 (0.22)
	R + 4	5.6 (3.4)	7.3 (4.5)	4.7 (2.9)	2.0 (1.4)	0.94 (0.88)	1.2 (1.1)	0.35 (0.29)
	R + 8	7.2 (5.0)	8.4 (5.7)	4.2 (2.8)	2.1 (1.5)	0.50 (0.40)	0.95 (1.0)	0.51 (0.32)
Pitch	Pre-flight	9.8 (6.6)	8.6 (4.9)	5.1 (3.3)	2.0 (1.3)	0.84 (0.46)	1.1 (1.0)	0.50 (0.54)
	R + 0	**5.1 (2.7)**	6.3 (5.7)	2.9 (2.5)	1.8 (1.6)	0.66 (0.48)	0.77 (0.56)	0.55 (0.66)
	R + 4	7.7 (5.8)	6.6 (5.2)	4.4 (3.8)	2.5 (2.2)	1.2 (1.2)	1.4 (1.6)	0.71 (0.77)
	R + 8	5.5 (4.7)	7.8 (5.4)	4.7 (3.8)	2.4 (1.6)	1.0 (0.8)	1.5 (1.2)	0.71 (0.50)
**SHADOW**								
Roll	Baseline	7.6 (7.2)	7.9 (5.0)	6.2 (4.6)	3.4 (2.8)	1.6 (2.9)	2.1 (2.1)	1.2 (1.2)
	G + 0	8.4 (3.9)	7.7 (1.5)	6.2 (2.4)	3.6 (1.8)	1.4 (0.7)	2.0 (1.5)	1.5 (1.7)
	G + 4	6.9 (2.3)	7.3 (3.0)	5.7 (2.9)	4.4 (2.3)	1.3 (0.9)	1.9 (1.8)	1.0 (0.9)
	G + 8	6.8 (2.6)	7.3 (3.4)	5.2 (2.2)	3.7 (2.5)	1.5 (0.0)	1.8 (1.6)	1.2 (1.1)
Pitch	Baseline	8.6 (5.0)	8.5 (3.7)	5.9 (3.6)	3.5 (2.7)	1.4 (0.8)	1.7 (1.2)	1.1 (0.8)
	G + 0	8.8 (3.9)	7.3 (3.0)	5.6 (3.5)	3.5 (1.5)	1.2 (0.7)	2.0 (1.3)	1.0 (0.6)
	G + 4	5.8 (3.9)	6.1 (3.0)	5.0 (2.2)	2.7 (1.6)	1.1 (0.7)	1.8 (1.0)	0.8 (0.7)
	G + 8	6.2 (3.3)	7.1 (3.1)	5.2 (4.0)	2.5 (1.0)	1.1 (0.5)	1.7 (1.0)	0.9 (0.7)

**Figure 6:**
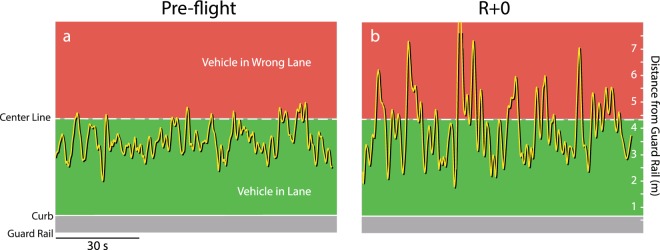
A comparison of pre-flight (**a**) and landing day (**b**) lane control during the mountain driving simulation in an astronaut subject.

**Figure 7:**
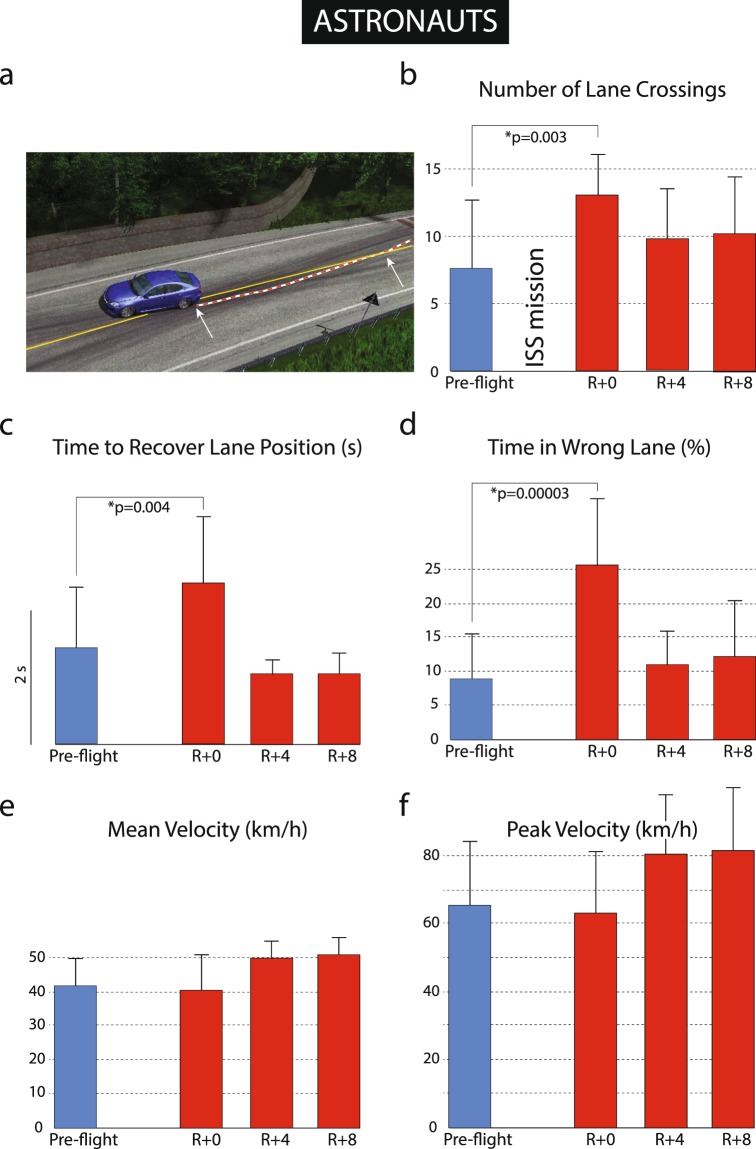
Performance on the 3 km mountain driving simulation for the astronaut group. (**a**) Lane deviations were assessed; (**b**) number of lane crossings, (**c**) time to recover lane position, (**d)** percent time in wrong lane, (**e)** mean and (**f)** peak vehicle velocity.

**Figure 8:**
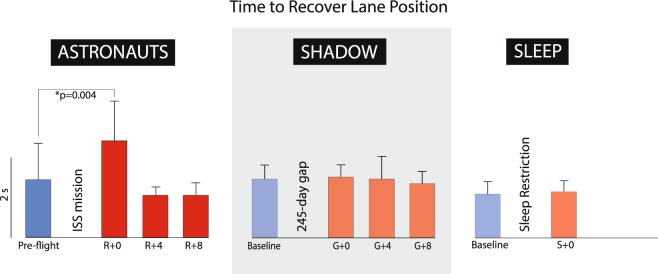
Time to recover lane position in the (**a**) astronaut, (**b**) shadow and (**c**) sleep-restricted groups.

There were profound deficits in post-landing driving performance for the 3 km winding road simulation in the astronaut group (Table 4). Pre-flight and landing day performance from one crewmember demonstrates a significant post-flight loss of vehicle control, with many more deviations onto the wrong side of the road (Fig. 6). This was consistent across the eight astronauts on R + 0, with significant increases in lane crossings (U = 35.5; p = 0.003; Fig. 7b), time to recover (U = 37; p = 0.004; Fig. 7c) and time spent in the wrong lane (U = 13; p = 0.00003; Fig. 7d). Astronaut driving performance recovered to baseline by four days after landing (R + 4). There were no changes in mountain driving performance in the shadow group or in the sleep group following the 30-h sleep restriction protocol (Table 4 and Fig. 8). There were no significant changes in driving performance on the cone driving task for the astronaut, shadow and sleep restriction groups (Table 5).

**Table 4:** Results from the mountain driving simulation. Significant changes shown in boldface.

ASTRONAUTS	PRE-FLIGHT	R + 0	R + 4	R + 8
Number of Lane Crossings	7.50 (5.21)	**12.88 (3.09)**	9.75 (3.77)	10.13 (4.29)
Time to Recover Lane Position (s)	1.41 (0.90)	**2.41 (1.00)**	1.05 (0.22)	1.04 (0.30)
Percent Time in Wrong Lane	8.60 (7.06)	**25.59 (9.81)**	10.90 (5.19)	12.09 (8.30)
Mean Velocity (km/h)	41.8 (8.5)	40.5 (10.4)	50.0 (5.1)	51.0 (5.3)
Peak Velocity (km/h)	65.9 (19.1)	63.6 (18.4)	80.0 (18.8)	82.2 (18.7)
**SHADOW**	**BASELINE**	**G + 0**	**G + 4**	**G + 8**
Number of Lane Crossings	15.06 (5.74)	16.41 (6.52)	13.92 (5.82)	14.75 (5.91)
Time to Recover Lane Position	1.30 (0.35)	1.37 (0.28)	1.37 (0.51)	1.21 (0.27)
Percent Time in Wrong Lane	21.93 (10.68)	25.46 (9.14)	21.38 (14.1)	20.68 (9.12)
Mean Velocity	53.6 (3.1)	54.5 (2.96)	54.0 (3.11)	55.4 (2.9)
Peak Velocity	89.2 (9.4)	92.4 (10.3)	88.5 (9.0)	95.6 (7.8)
**SLEEP**	**BASELINE**	**S + 0**		
Number of Lane Crossings	10.42 (4.96)	10.38 (3.62)		
Time to Recover Lane Position	1.09 (0.31)	1.14 (0.29)		
Percent Time in Wrong Lane	11.72 (6.78)	13.29 (7.55)		
Mean Velocity	48.9 (3.9)	50.9 (4.3)		
Peak Velocity	79.5 (13.2)	81.7 (13.2)

**Table 5:** Results from the cones driving simulation.

ASTRONAUTS	PRE-FLIGHT	R + 0	R + 4	R + 8
Number of Cones Hit	3.3 (2.4)	3.6 (2.8)	2.6 (1.6)	3.0 (2.7)
Time to Complete (s)	89.2 (12.6)	95.8 (23.2)	80.4 (7.01)	80.3 (8.5)
Mean Velocity (km/h)	61.7 (8.7)	58.8 (12.0)	67.7 (5.9)	67.9 (7.1)
**SHADOW**	**PRE-FLIGHT**	**G + 0**	**G + 4**	**G + 8**
Number of Cones Hit	3.1 (3.0)	3.2 (2.7)	3.4 (2.3)	3.2 (3.7)
Time to Complete (s)	81.9 (6.1)	83.7 (8.4)	79.0 (6.2)	78.5 (7.4)
Mean Velocity (km/h)	66.3 (4.9)	65.0 (5.9)	68.7 (5.2)	69.4 (6.4)
**SLEEP**	**PRE-FLIGHT**	**S + 0**		
Number of Cones Hit	3.0 (2.6)	4.0 (3.8)		
Time to Complete (s)	85.9 (7.9)	83.4 (10.9)		
Mean Velocity (km/h)	63.4 (5.7)	65.7 (8.7)		

## Discussion

### Cognitive/sensorimotor test battery

The results of this study demonstrate subtle but significant changes in cognitive/sensorimotor performance on the day of return (R + 0) from ISS missions that adversely affected operator proficiency. Self-reported sleepiness, unsurprisingly, was higher in the astronauts on landing day. There was a small but significant decline in manual dexterity, and manual tracking performance during dual tasking was significantly impaired. Although not conclusive, there was some evidence of a degradation in motion perception at frequencies below 0.43 Hz. None of these changes were observed in the shadow group. The sleep cohort, who reported a significant increase in sleepiness after the 30-h sleep restriction protocol analogous to the astronaut group, did not exhibit impaired tracking during dual tasking. These findings demonstrate that, with the exception of subjective sleepiness, the changes in performance on the cognitive/sensorimotor test battery observed in the astronaut group on R + 0 were likely due to factors associated with spaceflight, rather than simply fatigue alone or lack of practice. The mild sensorimotor effects (manual dexterity declined by around 10%) suggest a general post-flight malaise in motor function and motion perception. Adding a distracting task significantly impaired manual tracking performance, indicating post-flight limitations in available central processing resources in the astronauts; a lack of cognitive reserve apparent only when faced with competing tasks. In isolation, none of these spaceflight-induced changes suggest major cause for concern; however, as discussed below, taken together they significantly affected operator proficiency.

### Driving performance

The ability to maintain lane position during the mountain driving simulation was significantly impaired in astronauts on R + 0. Crewmembers made more crossings into the wrong lane, took longer to correct, and spent a greater percentage of time in the wrong lane compared to before flight. These changes were not observed in the shadow or sleep cohorts, which strongly suggest that the degradation in astronaut driving performance was related to spaceflight rather than fatigue alone.

Performance on the cone course was unaffected in all three cohorts. One possible explanation may be the different frequency characteristics of the mountain and cone driving tasks. Slaloming about the cones induced rapid changes in the orientation of the gravito-inertial acceleration (GIA) vector (the sum of gravity and centripetal acceleration, utilized by the brain to perceive tilt^40^, simulated by roll tilts of the cabin) in the coronal plane at a frequency of around 0.3 Hz. The large curves on the mountain course generated GIA tilts at much lower frequencies (<0.05 Hz). Motion perception was impaired at very low (tilt) frequencies in the astronaut group on landing day, thus it may have been harder for crewmembers to maintain a long sweeping curve on the mountain course as opposed to a rapid oscillation of the vehicle about the cones.

### Time course of recovery

Performance decrements in manual dexterity, dual tasking and motion perception observed in the astronaut group on R + 0, and subjective sleepiness, recovered by the second post-flight session (3–5 days post-landing). Lane control on the mountain driving course also recovered by R + 4.

### Possible causes of degradation in post-flight operator proficiency

The linear accelerometers of the inner ear, the otoliths, sense the orientation of the head with respect to gravity, and contribute to a range of physiological functions including balance, gaze, movement and perception of motion. Missions to the ISS place crewmembers in a microgravity environment where ‘tilt’ (in the vestibular sense of head position with regard to gravity) has no meaning. Several studies have documented in- and post-flight deficits in low-frequency (‘tilt’) otolith function (see^34^ for a review), including our recently published study of the ocular counter-rolling reflex (OCR). OCR is an otolith-mediated response that rolls the eyes in the opposite direction to roll head tilt on Earth, and is a direct measure of low-frequency otolith function. We found a significant post-flight reduction in OCR gain in 25 cosmonauts that persisted up to 5 days after return from the ISS^11^. The human tilt response is frequency limited to approximately 0.33 Hz^12^, and the motion perception results from the current study demonstrated evidence of a reduction in sensitivity to roll and pitch motions of the cabin at low frequencies, consistent with flight studies showing impairment in low-frequency otolith function. Post-flight blunting of the tilt response would affect operator proficiency on tasks that require perception of or alignment with tilted visual or inertial cues, such as the mountain driving course.

Fatigue is likely a factor in post-flight performance decrements, but the results of the sleep restriction study suggest that sleepiness alone was not responsible. Astronauts were sleepier on R + 0 but reaction time was unchanged, so subjects could attend to a task for short periods despite their fatigue. The impairment in manual dexterity perhaps reflects persistence of in-flight adaptation of fine motor control to microgravity that is maladapted to the terrestrial environment. However, the reduction in the number of pins placed on landing day was less than 10%, and it seems unlikely that the dramatic post-flight inability to maintain lane control was due primarily to ‘finger trouble’. The degradation in manual tracking during dual tasking points to post-landing limitations in cognitive processing resources that were unique to the astronaut cohort. The results do not support a vestibular basis for this post-flight dual-tasking impairment. Cognitive tasks that utilized cortical areas receiving vestibular input^32^, such as perspective taking (parietal-temporal junction and superior parietal lobule) and match-to-sample (hippocampus), were unaffected on R + 0 (Table 2). Moreover, performance on the same dual-tasking paradigm was unchanged by application of pseudorandom Galvanic vestibular stimulation in a recent ground study^32^.

The post-flight impairment of operator proficiency may also be related to environmental factors unique to spaceflight. The partial pressure of CO_2_ on the ISS, averaging slightly less than 4 mm Hg over a 7-day period^41^, is considerably higher than on Earth (0.3 mm Hg), and elevated CO_2_ levels have been linked to an increased probability of headache in ISS crewmembers^41^. However, current US Environmental Protection Agency guidelines state that the maximum exposure limit is indefinite at CO_2_ levels below 7.6 mm Hg^42^. The altered light-dark cycle, sleep deprivation, high mental and physical workload of astronauts, all experienced in a confined space, have been simulated in laboratory studies such as Mars520, in which 6 male subjects lived and worked in a 550 m^3^ facility (a little more than half the volume of the ISS) from June 3^rd^ 2010 until November 4^th^ 2011. Subjects were found to exhibit decreased cortical activity associated with sensory deprivation and monotony, and diminished sleep quality, although any functional impact on cognition and operator proficiency was not elucidated^43^. Similarly, studies of cognitive performance in personnel over-wintering in Antarctica found no significant changes^43^.

Taken together, the results suggest that a range of subtle physiological changes in spaceflight combine to leave astronauts vulnerable to performance decrements on the day of landing, particularly when faced with low-frequency GIA or visual tilts and multiple and competing task requirements. A striking feature of our results is the disparity between the subtle nature of the changes observed in astronaut performance on the cognitive/sensorimotor test battery on landing day and the profound impact these changes had on operator proficiency. It is difficult to imagine a direct causative link between any single test deficit observed in this study and the observed impairment in driving ability; say, for example, the 10% decrease in manual dexterity and the inability to maintain lane control. Moreover, it is also difficult to imagine that addressing any single deficit, such as an in-flight program that maintained manual dexterity at pre-flight levels, would significantly improve post-flight operator proficiency. Our results suggest that countermeasure development should target the cumulative effect of the subtle physiological changes observed on landing day, rather than focusing on individual cognitive or sensorimotor impairments. Based on our results, the following countermeasure recommendations were made to NASA’s Mission Architecture Team:

- In-flight high-fidelity ‘just-in-time’ refresher training for landing and post-flight manual control tasks
- Improved displays/non-visual aids to support crewmembers during manual control maneuvers with a tilt component (visual or gravito-inertial)
- Cognitive/sensorimotor self-assessments to gauge fitness for duty before conducting challenging manual control tasks
- Consider limitations in dual tasking when assigning crewmember assignments during critical stages of manual control

## Supplementary information

Preflight and landing day (R + 0) driving performance in an astronaut subject
